# Supplementary material for: Saccharomyces boulardii (CNCM I-745) improves intestinal damage in sepsis by remodeling intestinal flora structure
Source: Front Cell Infect Microbiol. 2025 Jul 31;15:1602792. doi: 10.3389/fcimb.2025.1602792 (PMC12350329; doi:10.3389/fcimb.2025.1602792)
Supplement: Supplementary file 1 [file DataSheet1.zip › summary/summary/7_Differential_analysis/2_LEfSe_result/Group_CW0_vs_AW0_0.05_3.0/LEfSe_Group_CW0_vs_AW0.abund.pdf]

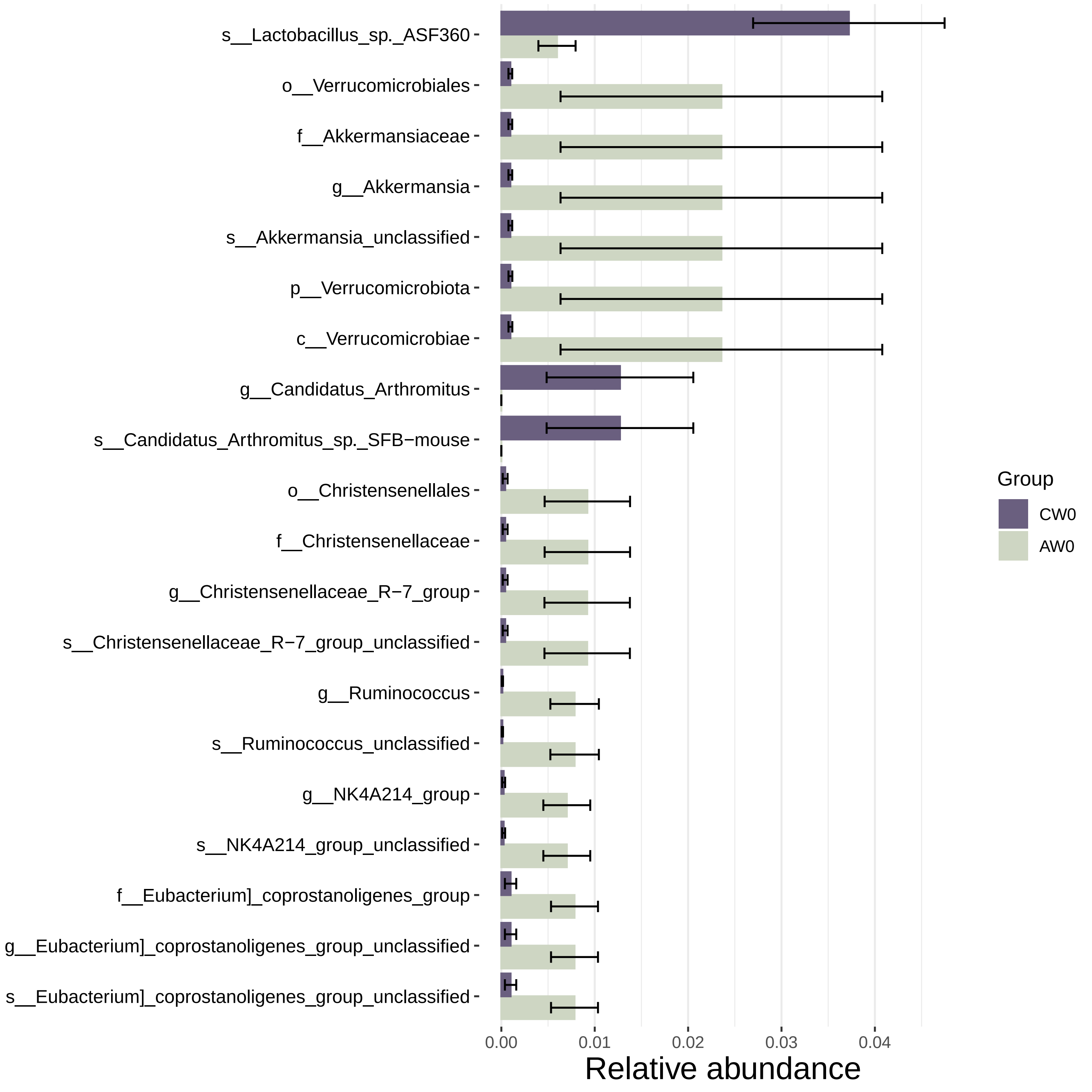

s__Lactobacillus_sp._ASF360
o__Verrucomicrobiales
f__Akkermansiaceae
g__Akkermansia
s__Akkermansia_unclassified
p__Verrucomicrobiota
c__Verrucomicrobiae
g__Candidatus_Arthromitus
s__Candidatus_Arthromitus_sp._SFB−mouse
o__Christensenellales
f__Christensenellaceae
g__Christensenellaceae_R−7_group
s__Christensenellaceae_R−7_group_unclassified
g__Ruminococcus
s__Ruminococcus_unclassified
g__NK4A214_group
s__NK4A214_group_unclassified
f__Eubacterium]_coprostanoligenes_group
g__Eubacterium]_coprostanoligenes_group_unclassified
s__Eubacterium]_coprostanoligenes_group_unclassified
0.00
0.01
0.02
0.03
0.04
Relative abundance
Group
CW0
AW0
